# ﻿Morphological and molecular evidence gives insight into the taxonomic position of *Peucedanumpubescens* (Apiaceae, Selineae)

**DOI:** 10.3897/phytokeys.213.89784

**Published:** 2022-11-09

**Authors:** Jiao-Jiao Deng, Chang-Kun Liu, Song-Dong Zhou, Xing-Jin He

**Affiliations:** 1 Key Laboratory of Bio-Resources and Eco-Environment of Ministry of Education, College of Life Sciences, Sichuan University, 610065, Chengdu, Sichuan, China Sichuan University Sichuan China

**Keywords:** Apiaceae, *Ligusticopsispubescens*, new combination, *Peucedanumpubescens*

## Abstract

In this study, morphological and molecular evidences were combined to determine the taxonomic position of *Peucedanumpubescens* Hand.-Mazz. Morphologically, *Peucedanumpubescens* is similar to the species of the genus *Ligusticopsis* in having fibrous remnant sheaths at the stem base, pinnate and linear coexisted bracts, strongly compressed dorsally mericarps, filiform median and lateral ribs, winged marginal ribs, numerous vittae in each furrow and commissure, but can also be easily distinguished from members of *Ligusticopsis* by its hispid fruit and linear-lanceolate bracteoles. Molecular phylogenetic analyses based on the single-copy protein-coding sequences (CDS) of plastomes and internal transcribed space (ITS) region showed that *Peucedanumpubescens* nested in the genus *Ligusticopsis*. As both morphological and molecular data supported the inclusion of *Peucedanumpubescens* within *Ligusticopsis*, the species is here transferred and the new combination, *Ligusticopsispubescens* (Hand.-Mazz.) J.J.Deng, C.K.Liu & X.J.He, made.

## ﻿Introduction

*Peucedanum* sensu lato was previously characterized by dorsally compressed mericarps with slightly prominent dorsal ribs, narrowly winged lateral ribs, as well as a broad commissure (Sheh 1992; Spalik et al. 2004; Sheh and Watson 2005). As such it was one of the largest genera of Apiaceae, comprising 100–120 species with 33 endemics to the territory of China, and with a distribution in Eurasia, South Africa, and sometimes Australia (Spalik et al. 2004; Sheh and Watson 2005; Cieśla et al. 2009). However, *Peucedanum* sensu lato has been a taxonomically confusing genus due to its great heterogeneous characteristics (Solov’eva et al. 1985; Reduron et al. 1997; Downie et al. 2000, 2010; Winter et al. 2008; Zhou et al. 2014), exhibiting a wide variety of life forms, leaf and fruit structures, and chemical compositions (Shneyer et al. 2003). Moreover, several molecular phylogenetic studies based on DNA fragments and plastomes indicated that *Peucedanum* sensu lato was not a monophyletic group (Downie et al. 2000; Spalik et al. 2004; Valiejo-Roman et al. 2006; Feng et al. 2009; Zhou et al. 2009, 2020; Liu et al. 2022). Consequently, the circumscription of the genus has been greatly reduced with *Peucedanum* sensu stricto, recognized by ternate leaves, linear-subulate or filiform bracteoles, one vitta in each furrow and two vittae on commissure in mericarp (Kadereit and Bittrich 2018) and several members of *Peucedanum* sensu lato were separated to restitute or establish genera or transfer into other genera (Reduron et al. 1997; Winter et al. 2008; Ostroumova et al. 2016; Pimenov et al. 2016; Pimenov 2017). However, the previous studies mainly focused on those species distributed in Europe and South Africa, and the taxonomic position of Chinese endemic species of this genus was still unresolved.

*Ligusticopsis* Leute was described by Leute in 1969 with *Ligusticopsisrechingeriana* Leute as its type species. The taxonomy of genus has been controversial since its establishment, due to its close morphology to *Ligusticum* (Zhou et al. 2008, 2009; Sun et al. 2010); e.g. Flora Reipublicae Popularis Sinicae and Flora of China treated *Ligusticopsis* as the synonym of *Ligusticum* and “*Ligusticum* in the broad sense”, respectively (Sheh 1992; Sheh and Watson 2005), whereas the genus was recognized by Pimenov et al. (2001, 2003). Recently, a phylogenetic study based on morphological and molecular data confirmed the monophyly of *Ligusticopsis* and nine “true species of *Ligusticopsis*” were recognized (Li et al. 2022); the members of the genus are characterized by the following diagnostic characters: stem base clothed in fibrous remnant sheaths, pinnate bracts, pinnate bracteoles longer than rays of umbellule, mericarps strongly compressed dorsally, median and lateral ribs filiform or keeled, marginal ribs winged, and numerous vittae in each furrow and commissure.

*Peucedanumpubescens* Hand.-Mazz. (1933: 728) was described based on a collection (E00002620) from Yunnan, China, and was an endemic species to China (Sheh and Watson 2005; Pimenov 2017). Due to dorsally compressed mericarps with slightly prominent dorsal ribs and narrowly winged lateral ribs, *P.pubescens* was recognized as a member of *Peucedanum* sensu lato (Handel-Mazzetti Heinrich 1933). However, after examination of the type specimen and protologue, field observation, and morphological and micro-morphological research into it, we found this species was characterized by stem base clothed in fibrous remnant sheaths, pinnate leaves, linear and pinnate coexisted bracts, strongly dorsally compressed fruits, numerous vittae in each furrow and commissure, and these features are significantly similar to members of *Ligusticopsis*. To determine the taxonomic position of *Peucedanumpubescens*, we performed morphological and molecular analyses.

## ﻿Materials and methods

### ﻿Morphological observation

The morphological features of *Peucedanumpubescens* were observed in field. Then, mericarp of this species was observed and photographed using a stereomicroscope, Nikon SMZ 25 (Japan). Furthermore, morphological diagnoses of nine “true species of *Ligusticopsis*” were obtained from type specimens from K, P, E, WU, BM, GH, KUN, and HNWP, Flora of China (Sheh and Watson 2005), and analysis performed by Li et al. (2022). The Herbarium code refers to Thiers (2015).

### ﻿DNA extraction, ITS amplifying and sequencing

Total genomic DNA was extracted from silica-dried leaves with plant genomic DNA kit (Cwbio Biosciences, Beijing, China). The universal primers ITS4 (5’-TCC TCC GCT TAT TGA TAT GC-3’) and ITS5 (5’-GGA AGT AAA AGT CGT AAC AAG G-3’, White et al. 1990) were used to amplify the entire internal transcribed sequences (ITS). Amplification was undertaken using a volume of 30 µl with 15 µl 2 × Taq MasterMix (CWBIO, China), 10 µl ddH2O, 1.5 µl forward primer, 1.5 µl reverse primer, and 2 µl total DNA. The amplification of the ITS region was obtained by initial denaturation for 3 min at 94 °C, followed by 30 cycles of 45 s at 94 °C, 70 s at 54 °C, and 90 s at 72 °C, and then a final extension of 10 min at 72 °C. All PCR products were separated using a 1.5% (w/v) agarose TAE gel and sent to Sangon (Shanghai, China) for sequencing.

### ﻿Plastome sequencing and assembly

The extracted total DNA was fragmented into 400 bp to construct the pair-end library, following the manufacturer’s protocol (Illumina, San Diego, CA, USA). The DNA libraries were sequenced on the Illumina NovaSeq platform at Personalbio (Shanghai, China). Quality control of the raw reads was performed using fastP v0.15.0 (-n 10 and -q 15) (Chen et al. 2018), produced at least 5GB clean reads per species. De novo genome assembly from the clean data was accomplished utilizing NOVOPlasty v2.6.2 (Dierckxsens et al. 2017), with a kmer length of 39 bp and a sequence fragment of the *rbcL* gene from *Ligusticopsisbrachyloba* (Franch.) Leute (Genebank no. MN204661) as the seed sequence. The assembled complete plastome was annotated initially by using PGA (Qu et al. 2019) and then examined using Geneious v9.0.2 (Kearse et al. 2012).

### ﻿Phylogenetic analyses

To confirm the phylogenetic position of *Peucedanumpubescens*, phylogenetic trees were reconstructed based on single-copy protein-coding sequences (CDS) of 34 plastomes and 36 ITS sequences (Table 1). *Chamaesiummallaeanum* Farille & S. B. Malla and *Chamaesiumviridiflorum* (Franch.) Wolff ex Shan were selected as outgroups according to the result of a previous study (Li et al. 2022). Plastome CDs and ITS sequences were respectively aligned using MAFFT v7.221 (Katoh and Standley 2013), and then manually adjusted in MEGA7.0 (Kumar et al. 2016) to obtain plastome CDs and ITS datasets. The two alignments were subjected to Maximum-Likelihood (ML) analyses and Bayesian Inference (BI). For ML analyses, the software RAxML v8.2.8 (Stamatakis 2014) was used to construct the phylogenetic trees with the GTR+G+I model and 1000 bootstrap (BS) replicates. Bayesian inference (BI) analyses were conducted by MrBayes version 3.2.7 (Ronquist et al. 2012) with the best-fit substitution model (GTR+G+I) determined by Modeltest v3.7 (Posada and Crandall 1998). Markov Chain Monte Carlo (MCMC) search was performed for 1 × 10^6^ generations, sampling every 100 generations. The first 25% of trees were discarded as burn-in and the remainder was used to generate the consensus tree. Results of phylogenetic analyses were visualized and edited in FigTree v1.4.2 (Rambaut and Drummond 2015).

**Table 1.** Voucher details and GenBank accession numbers of taxa used in this study. A n-dash (–) indicates unavailable information.

Species	Voucher information	Locality	GenBank accession number	
			Plastome	ITS
*Angelicacartilaginomarginata*	13E-39-3, SZ2006071804	USA, NIH; China, Yunnan	NC029393	EU647210
*Angelicdecursiva*	13Q-02-1; v20060825 (SZ)	Korea, Cheongju-si; China, Sichuan	KT781591	EU418375
*Angelicagigas*	13E-39-3; SZ744110	USA, NIH; China, Sichuan	NC029393	GU395156
*Angelicalaxifoliata*	–; 2006071804 (SZ)	China, Sichuan; China, Yunnan	NC040122	EU647210
*Angelicanitida*	–; 2006080501 (SZ)	China, Qinghai; China, Sichuan	MF594405	EU418378
*Bupleurumchinense*	–; C.Q. Feng	China, Sichuan	MN893666	EU001334
*Bupleurumcommelynoideum*	–; 2008082002 (SZ)	China, Sichuan	MN893666	GU269874
*Chamaesiummallaeanum*	–; Strain NLM	China, Sichuan; China, Xizang	MT162552	KY74426
*Chamaesiumviridiflorum*	–; Strain HB	China, Yunnan	MN119373	KY744260
*Glehnialittoralis*	–; SZ666775	China, Sichuan	KU921430	GU395183
*Hanseniaforbesii*	–; SZ666939	China, Sichuan; China, Yunnan	NC034645	GU390407
*Hanseniaoviformis*	–; F22	China, Sichuan	MT843761	MT337430
*Hanseniaweberbaueriana*	–; J18091701	China, Sichuan	MN049520	MN049520
*Ligusticopsisbrachyloba*	L081401 (SZ); L0814 (SZ)	China, Chongqing	MZ491174	MZ497218
*Ligusticopsiscapillacea*	RT2019100601 (SZ); XB	China, Yunnan	NC049051	MT974023
*Ligusticopsishispida*	RT2019100301 (SZ); L08110501 (SZ)	China, Yunnan	NC049052	OL600824
*Ligusticopsisintegrifolia*	RT2019100202 (SZ); L081003 (SZ)	China, Yunnan	NC049055	MZ497219
*Ligusticopsisinvolucrata*	PC2018101905 (SZ); DB14	China, Yunnan	NC049054	MT974014
*Ligusticopsismodesta*	L081903 (SZ); L08190301 (SZ)	China, Yunnan	OL547615	OL600822
*Ligusticopsispubescens*1	LCK2020817001	China, Yunnan	ON872189	ON870396
*Ligusticopsispubescens*2	LCK2020817002	China, Yunnan	–	ON870397
*Ligusticopsispubescens*3	LCK2020817003	China, Yunnan	–	ON870398
*Ligusticopsisrechingeriana*	L081103 (SZ); L081103 (SZ)	China, Yunnan	MZ491175	MZ497220
*Ligusticopsisscapiformis*	RT2019082001 (SZ); CT9	China, Sichuan	NC049057	MT974012
*Ligusticopsiswallichiana*	LD081506 (SZ); LD08150601 (SZ)	China, Xizang	OL547616	OL600823
*Ligusticumdelavayi*	RT2019100301 (SZ); L08110501 (SZ)	China, Yunnan	NC049052	OL600824
*Ligusticumjeholense*	–; LGB1	China, Liaoning; China, Beijing	MT561037	KJ999437
*Ligusticumsinense*	–; Ge131139	China, Sichuan; China, Guangdong	NC038088	MH712648
*Ligusticumtenuissimum*	13I-08; JKTM-1-000065	Korea, Cheongju-si; Korea, Anyang-myeon	NC029394	KP058314
*Ligusticumthomsonii*	RT2019082301 (SZ); CJ (SZ)	China, Sichuan	MT409619	MT974009
*Meeboldiayunnanensis*	–; G18071908	China, Yunnan	MK993275	MN688997
*Peucedanumampliatum*	JQP19082505 (SZ); NASLQX022	China, Shanxi; –	OK336475	JF977799
*Peucedanumdelavayi*	SZ YY 062105; YY062105 (SZ)	–	EU418386	MT843765
*Peucedanumjaponicum*	LCK2020001 (SZ); sb1	China, Sichuan; –	OK336477	EU224273
*Peucedanummedicum*	LCK2020004 (SZ); SZ66876	China, Guangxi; –	OK336473	HQ256686
*Peucedanumpraeruptorum*	–; SZ ZXM 001	–	MN016968	EU418383

## ﻿Results

### ﻿Morphological comparison

*Peucedanumpubescens* is similar to the species of *Ligusticopsis* in having fibrous remnant sheaths at the stem base (Fig. 3B), pinnate leaves (Fig. 3A, C–E), linear and pinnate coexisted bracts (Fig. 3F), strongly dorsally compressed fruits (Fig. 3G, H), numerous vittae in each furrow and commissure (Fig. 3H), but can be easily distinguished from the latter by the morphological characters shown in Table 2. In detail, *Peucedanumpubescens* is different from *L.rechingeriana* Leute, *L.involucrata* (Franch.) Lavrova and *L.hispida* (Franch.) Lavrova et Kljuykov in that *P.pubescens* has a triangular-ovate leaf blade in outline (Fig. 3C, D) (vs. oblong-ovate or lanceolate), obovate ultimate segments (Fig. 3C) (vs. ovate or linear), pinnate and linear coexist bracts (Fig. 3F) (vs. pinnate), linear-lanceolate bracteoles (vs. pinnate) (Fig. 3F), ovate to obovate mericarp shape (Fig. 3G) (vs. elliptic to ovate or elliptic), hispid mericarp surface (Fig. 3G, H) (vs. smooth) and 2–3 vittae in each furrow (Fig. 3H) (vs. 1–3). *Peucedanumpubescens* can be distinguished from *L.integrifolia* (H. Wolff) Leute, *L.brachyloba* and *L.modesta* (Diels) Leute in having linear-lanceolate bracteoles (Fig. 3F) (vs. pinnate and linear coexist or pinnate), hispid mericarp surface (Fig. 3G, H) (vs. smooth), ovate or obovate to orbicular mericarp shape (Fig. 3G) (vs. elliptic to ovate or elliptic) and 2–3 vittae in each furrow (Fig. 3H) (vs. 1–3 or 3–4 in each furrow). *Peucedanumpubescens* differs from *L.capillacea* Leute and *L.scapiformis* (H. Wolff) Leute in having triangular-ovate leaf blade in outline (Fig. 3C, D) (vs. oblong-ovate or oblong-lanceolate), pinnate and linear coexist bracts (Fig. 3F) (vs. pinnate), linear-lanceolate bracteoles (Fig. 3F) (vs. pinnate) and hispid mericarp surface (Fig. 3G, H) (vs. smooth). *Peucedanumpubescens* can be distinguished from *L.wallichiana* (DC.) Pimenov et Kljuykov in having pinnate and linear coexist bracts (Fig. 3F) (vs. pinnate), linear-lanceolate bracteoles (Fig. 3F) (vs. pinnate and linear coexist), ovate or obovate to orbicular mericarp shape (Fig. 3G) (vs. elliptic), hispid mericarp surface (Fig. 3G, H) (vs. smooth), 2–3 vittae in each furrow (Fig. 3H) (vs. 1–3) and filiform median rib shape (Fig. 3G, H) (vs. keeled).

**Table 2.** Comparison of morphological characteristics between *Peucedanumpubescens* and the species of *Ligusticopsis*.

Characteristics	*P.pubescens*	*L.rechingeriana*	*L.involucrata*	*L.hispida*	*L.integrifolia*	*L.brachyloba*	*L.modesta*	*L.capillacea*	*L.scapiformis*	*L.wallichiana*
**Stem (base)**	Fibrous remnant	Fibrous remnant	Fibrous remnant	Fibrous remnant	Fibrous remnant	Fibrous remnant	Fibrous remnant	Fibrous remnant	Fibrous remnant	Fibrous remnant
**Leaves**	Pinnate, triangular-ovate	Pinnate, oblong-ovate	Pinnate, oblong-ovate	Pinnate, lanceolate	Pinnate, oblong-ovate	Pinnate, triangular-ovate	Pinnate, oblong-ovate	Pinnate, oblong-ovate	Pinnate, oblong-lanceolate	Pinnate, broadly ovate
**Ultimate segments of leaves**	Obovate	Ovate	Linear	Linear	Oblong-ovate or lanceolate	Oblong-ovate or lanceolate	Linear or lanceolate	Obovate	Ovate	Linear
**Bracts**	Pinnate and linear coexist	Pinnate	Pinnate	Pinnate	Pinnate and linear coexist	Pinnate	Pinnate	Pinnate	Pinnate	Pinnate
**Bracteoles**	linear-lanceolate	Pinnate	Pinnate	Pinnate	Pinnate and linear coexist	Pinnate	Pinnate	Pinnate	Pinnate	Pinnate and linear coexist
**Mericarp surface**	Hispid	Smooth	Smooth	Smooth	Smooth	Smooth	Smooth	Smooth	Smooth	Smooth
**Mericarp shape**	ovate or obovate to orbicular	Elliptic to ovate	Elliptic	Elliptic	Elliptic to ovate	Elliptic	Elliptic to oblong	Ovate	Elliptic to ovate	Elliptic
**Calyx teeth**	Conspicuous	Conspicuous	Conspicuous	Conspicuous	Conspicuous	Conspicuous	Conspicuous	Conspicuous	Conspicuous	Conspicuous
**Dorsal compression**	Strong	Strong	Strong	Strong	Strong	Strong	Strong	Strong	Strong	Strong
**Median rib shape**	Filiform	Filiform	Filiform	Filiform	Filiform	Keeled	Filiform	Filiform	Filiform	Keeled
**Vittae each furrow**	2–3	1–3	1–3	1–3	1–3	2–3	3–4	1–3	1–4	1–3
**Commissural vittae**	6	6	6	6	6	6	8	6	4–6	6

### ﻿Plastome feature of *Peucedanumpubescens*

**Figure 1.**
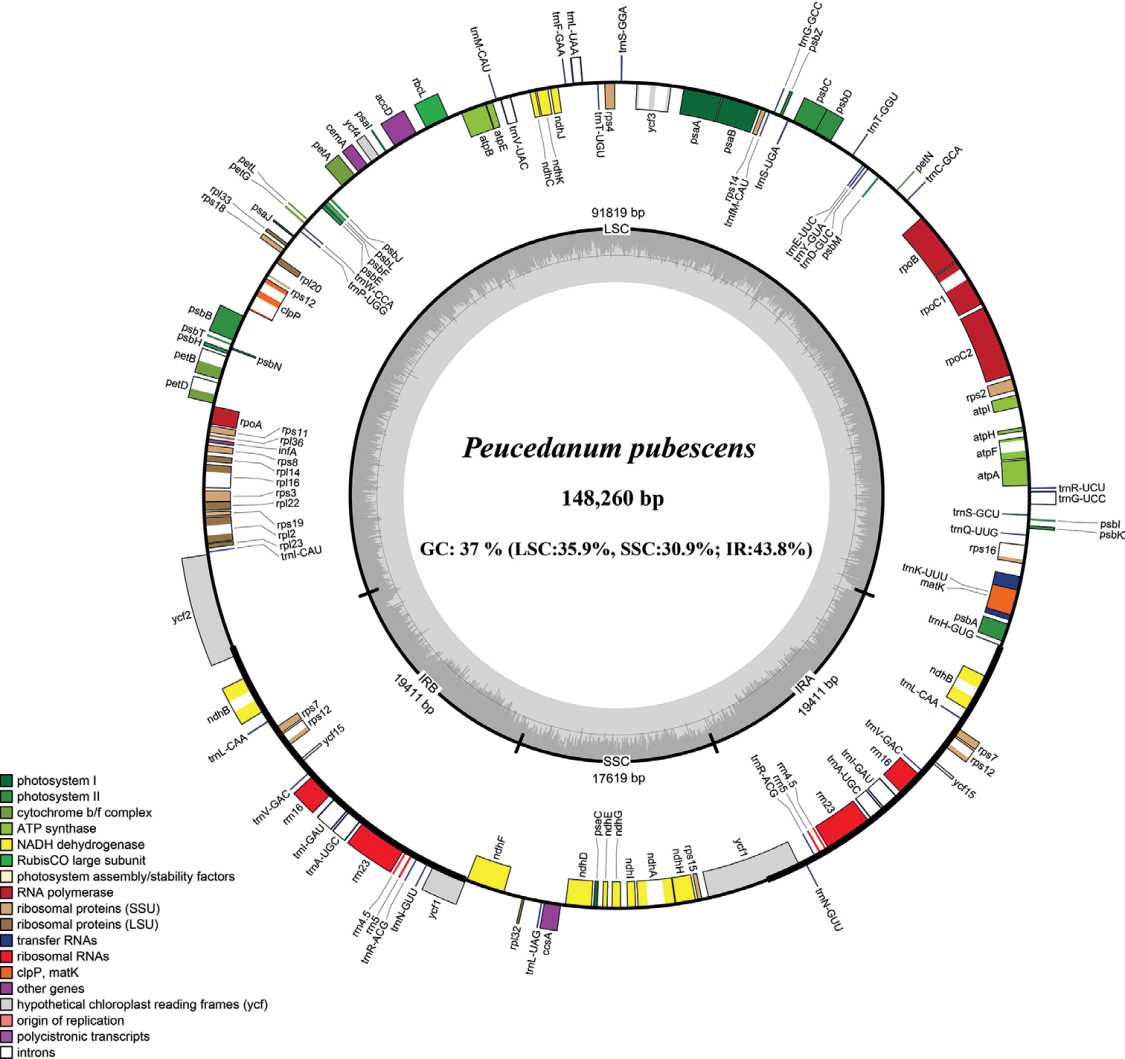
Plastome map of *Peucedanumpubescens*.

The plastome of *Peucedanumpubescens* is a typically quadripartite structure, including a large single copy region (LSC), a small single copy region (SSC), and a pair of inverted repeat regions (IR) (Fig. 1). The overall size of plastome is 148,260 bp, and that of the LSC, IR, and SSC are 91,819 bp, 19,411 bp, and 17,619 bp, respectively. GC content analysis shows that the overall GC content is 37.0%, and the IR regions (43.8%) are higher than LSC (35.9%) and SSC (30.9%). The whole plastid genome contains 129 genes including 36 tRNAs, 8 rRNAs, and 85 protein-coding genes.

### ﻿Phylogenetic analyses

The phylogenetic trees based on plastome CDs and ITS were given in Fig. 4 and Fig. 5, respectively. Both tree topologies strongly supported that *Peucedanumpubescens* nested in the genus *Ligusticopsis* (PP = 1.00 & BS = 100%; PP = 0.99 & BS = 88%). Although the phylogenetic position of this species could not be resolved in ITS tree, phylogenetic tree constructed based on plastome CDs showed that *Peucedanumpubescens* was sister to the clade that included the species *L.rechingeriana* (type species of the genus *Ligusticopsis*) and *L.involucrata* with high support (PP = 1.00 & BS = 99%).

## ﻿Discussion

*Peucedanum* sensu stricto and *Ligusticopsis* both belong to the Selineae tribe of Apiaceae, and members of these two genera are similar in the dorsally compressed fruits with filiform dorsal ribs, and winged marginal ribs (Spalik et al. 2004; Sheh and Watson 2005; Li et al. 2022), but the former genus can be distinguished significantly from the latter by having ternate leaves, linear-subulate, caducous or lacking bracts, one vitta in a furrow and two vittae in commissure in mericarp (Kadereit and Bittrich 2018), while the latter can also be distinguished from the former by possessing pinnate leaves, pinnate bracts, numerous vittae in each furrow and in commissure (Li et al. 2022). *Peucedanumpubescens* is more similar to the genus *Ligusticopsis* in having pinnate leaves, linear and pinnate coexisting bracts, numerous vittae in each furrow and in commissure (Table 2), rather than *Peucedanum* sensu stricto. This result was further supported by the molecular phylogenetic analyses that *Peucedanumpubescens* nested in *Ligusticopsis*. As a result, *Peucedanumpubescens* is here transferred to *Ligusticopsis* as an independent species and a new combination in *Ligusticopsis* made, so that this genus now includes ten recognized species. The species is easily distinguished from other members of *Ligusticopsis* by the hispid fruit and linear-lanceolate bracteoles.

### ﻿Taxonomic treatment

#### ﻿ Ligusticopsis pubescens

Taxon classificationPlantaeApialesApiaceae

(Hand.-Mazz.) J.J.Deng, C.K.Liu & X.J.He comb. nov.

40B36C04-B2D0-5843-90D2-217B5F1E743B

urn:lsid:ipni.org:names:77307802-1

Figs 2 , 3

##### Basionym.

*Peucedanumpubescens* Hand.-Mazz. (1933: 728).

##### Type.

China. Yunnan centralis: In regionis calide temperatae ad orientem fluminis Dsolin-ho, declivibus siccis inter vicos Mabou schan et Bölu, ad elevationem 1900–2000 m, 9 November 1916, Handel-Mazzetti 13043 (lectotype: WU! (WU0029560); isolectotypes: E (E00002620), W!).

##### Description.

Perennials. Plants 30–70 cm, densely pubescent throughout. Stem solitary, hollow, prominent striated protrusions, branches few, short and stout, base densely clothed with fibrous leaf remains. Basal leaves few; petioles with broadly scarious-margined sheaths; leaf blade triangular-ovate in outline, 8–10 × 8–10 cm, 1–2-pinnate, pinnae sessile or subsessile; ultimate segments obovate, 1–4.5 × 0.8–2 cm, rather thick, both surfaces tomentose, more densely so on abaxial nerves, coarsely serrate or crenate, base cuneate or truncate. Leaves reduced upwards, uppermost very small, 3-lobed or toothed, petioles wholly sheathing. Synflorescence subcorymbosely branched; umbels 2.5–4 cm across; peduncles angled; bracts 6 to 8, pinnate and linear coexist, hispid throughout; rays 10 to 15, subequal, 1–2 cm; bracteoles 5 to 7, linear-lanceolate, longer than flowers; umbellules ca. 10-flowered. Calyx teeth conspicuous, subulate. Petals white, stylopodium conical, styles long, ca. 2 mm. Fruit ovate or obovate to orbicular, ca. 4 × 3 mm, hispid; strong dorsal compression, vittae large, 2–3 in each furrow, 6 on commissure. Seed face plane.

**Figure 2.**
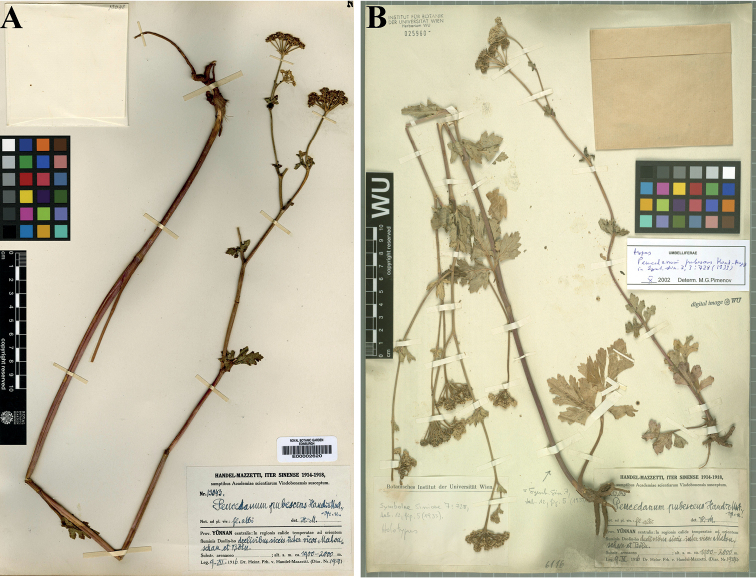
*Peucedanumpubescens***A** isolectotype (E00002620) **B** lectotype (WU0029560).

**Figure 3.**
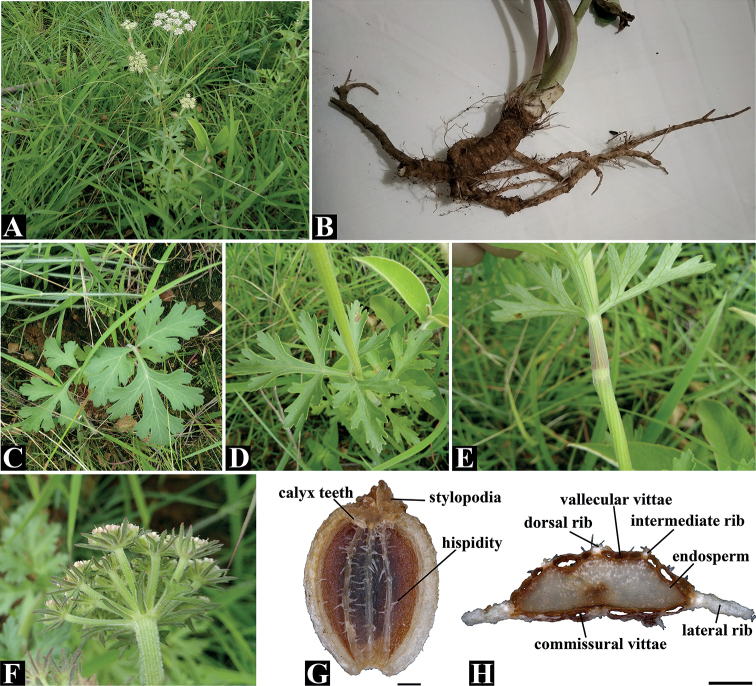
*Peucedanumpubescens***A** habit **B** root **C** basal leaf **D** middle leaf **E** middle leaf with scarious-margined sheaths **F** bracts and bracteoles **G** dorsal view of mericarp **H** transverse section of mericarp. Scale bars: 0.5 mm (**G, H**).

##### Phenology.

Flowering and fruiting: August to October.

##### Vernacular name.

Máo qián hú (Chinese pronunciation), 毛前胡 (Chinese name).

##### Distribution and habitat.

This species is endemic to China and distributed in Yunnan (Lufeng, Luquan, Wuding) and Sichuan (Huili, Miyi) provinces. It grows in alpine meadows, elevation 1900–3000 m.

##### Diagnosis.

*Ligusticopsispubescens* significantly differs from other *Ligusticopsis* species by linear-lanceolate bracteoles and hispid fruit.

##### Additional specimen examined.

China. Sichuan: Huili, Hongge, 2200 m, 12 October 1958, *Z. He*, *S.G*. *Tang* & *B.Q*. *Li 11593* (NAS); Panzhihua, Baishapo, 26°35'17"N, 101°59'1"E, 1854 m, 17 August 2021, *C.K*. *Liu LCK2020817001* (SZ).

### ﻿Key to related species

**Table:** 

1	Fruit hispid	***L.pubescens***
–	Fruit smooth	**2**
2	Bracteoles pinnate and linear coexist	**3**
–	Bracteoles pinnate	**4**
3	Blade shape broadly ovate; calyx teeth linear	***L.wallichiana***
–	Blade shape triangular-ovate; calyx teeth lanceolate	***L.brachyloba***
4	Bracts pinnate and linear coexist; petals white obcordate	***L.integrifolia***
–	Bracts pinnate; petals white purplish obcordate	**5**
5	Commissural vittae 8	***L.modesta***
–	Commissural vittae 4 to 6 or 6	**6**
6	Plants hispid throughout	**7**
–	Plants sparsely pilose or glabrous	**8**
7	Rays extremely elongated, elongate up to 24 cm; calyx teeth linear	***L.hispida***
–	Rays subequal, (1–)3 cm; calyx teeth lanceolate	***L.capillacea***
8	Stem unbranched; vittae per furrow 1 to 4, commissural vittae 4 to 6	***L.scapiformis***
–	Stem usually branched; vittae per furrow1 to 3, commissural vittae 6	**9**
9	Ultimate leaf segments oblong-ovate; mericarp elliptic	***L.involucrata***
–	Ultimate leaf segments ovate; mericarp elliptic to ovate	***L.rechingeriana***

**Figure 4.**
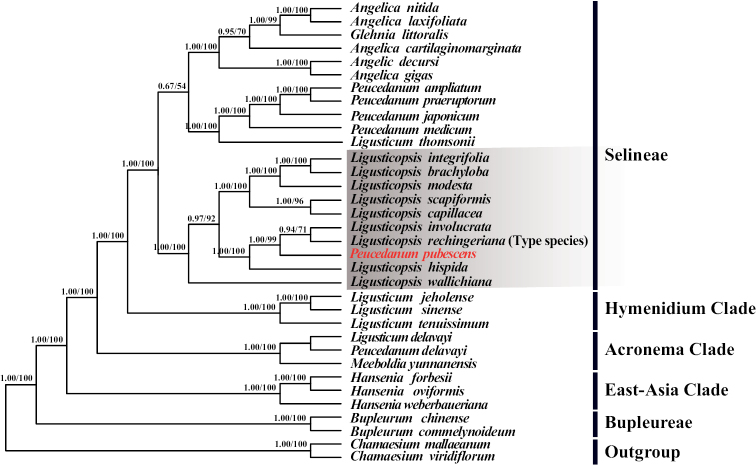
Phylogenetic tree inferred from Maximum-Likelihood (ML) and Bayesian Inference (BI) analyses based on 79 commonly shared CDs. PP/BS indicated posterior probabilities/bootstrap values.

**Figure 5.**
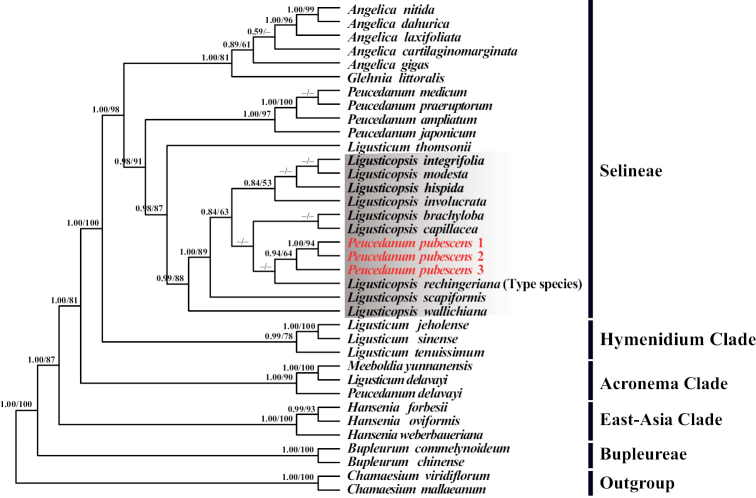
Phylogenetic tree of *Peucedanumpubescens* inferred from Maximum-Likelihood (ML) and Bayesian Inference (BI) analyses based on 36 ITS sequences. PP/BS indicated posterior probabilities/bootstrap values, respectively. Short line indicates values < 50%.

## Supplementary Material

XML Treatment for Ligusticopsis pubescens
